# People living in hilly residential areas in metropolitan Perth have less diabetes: spurious association or important environmental determinant?

**DOI:** 10.1186/1476-072X-12-59

**Published:** 2013-12-21

**Authors:** Karen Villanueva, Matthew Knuiman, Mohammad Javad Koohsari, Sharyn Hickey, Sarah Foster, Hannah Badland, Andrea Nathan, Fiona Bull, Billie Giles-Corti

**Affiliations:** 1 Centre for the Built Environment and Health, School of Population Health, The University of Western Australia, 35 Stirling Highway, Crawley, Western Australia 6009, Australia; 2 School of Population Health, The University of Western Australia, 35 Stirling Highway, Crawley, Western Australia 6009, Australia; 3 Behavioural Epidemiology Laboratory, Baker IDI Heart and Diabetes Institute, 75 Commercial Road, Melbourne, Victoria 3004, Australia; 4 McCaughey VicHealth Centre for Community Wellbeing, Melbourne School of Population and Global Health, University of Melbourne, Level 5, 207 Bouverie Street, Melbourne, Victoria 3010, Australia; 5 School of Public Health and Social Work, Queensland University of Technology, Kelvin Grove Campus, Brisbane, Queensland 4006, Australia

**Keywords:** Adults, Built environment, Diabetes, Hilly, Neighbourhood, Slope, Terrain, Walking

## Abstract

**Background:**

Variations in ‘slope’ (how steep or flat the ground is) may be good for health. As walking up hills is a physiologically vigorous physical activity and can contribute to weight control, greater neighbourhood slopes may provide a protective barrier to weight gain, and help prevent Type 2 diabetes onset. We explored whether living in ‘hilly’ neighbourhoods was associated with diabetes prevalence among the Australian adult population.

**Methods:**

Participants (≥25 years; n = 11,406) who completed the Western Australian Health and Wellbeing Surveillance System Survey (2003–2009) were asked whether or not they had medically-diagnosed diabetes. Geographic Information Systems (GIS) software was used to calculate a neighbourhood mean slope score, and other built environment measures at 1600 m around each participant’s home. Logistic regression models were used to predict the odds of self-reported diabetes after progressive adjustment for individual measures (i.e., age, sex), socioeconomic status (i.e., education, income), built environment, destinations, nutrition, and amount of walking.

**Results:**

After full adjustment, the odds of self-reported diabetes was 0.72 (95% CI 0.55-0.95) and 0.52 (95% CI 0.39-0.69) for adults living in neighbourhoods with moderate and higher levels of slope, respectively, compared with adults living in neighbourhoods with the lowest levels of slope. The odds of having diabetes was 13% lower (odds ratio 0.87; 95% CI 0.80-0.94) for each increase of one percent in mean slope.

**Conclusions:**

Living in a hilly neighbourhood may be protective of diabetes onset or this finding is spurious. Nevertheless, the results are promising and have implications for future research and the practice of flattening land in new housing developments.

## Background

Preventing and managing Type 2 diabetes is one of the fastest growing global public health concerns of our time [1]. In 2007–08, an estimated 818,200 Australians (4% of the population) reported medically diagnosed diabetes (excluding gestational), of which the majority (96%) was Type 2 diabetes [2]. This represents a 1.13% increase since the 2004–05 Australian National Health Survey [3]. On a global scale it is estimated that the number of adults aged 20 years or more with Type 2 diabetes will double from 171 million in 2000 to 266 million in 2030 (in [1]). Type 2 diabetes is generally considered a ‘lifestyle disease’, with onset typically occurring in middle to late adulthood [3]. It is well known that physical inactivity, unhealthy diets and obesity are important lifestyle risk factors for Type 2 diabetes [4-7]. These represent major modifiable risk factors in reducing morbidity and mortality from Type 2 diabetes [8,9].

Where we live influences our physical activity, exercise habits and diet choices, by providing access to healthy and unhealthy food options, local recreational and utilitarian destinations and public transport [10]. There is growing evidence that the way neighbourhoods are designed (i.e., the built environment) influences different types of physical activity, including walking and cycling [10-14]. Walking, is a popular, versatile, and potentially enjoyable outdoor activity that is recognised as a means of increasing levels of physical activity for the majority of the able-bodied population, particularly if done briskly [15]. However, while the relationship between the built environment (e.g., street connectivity, traffic exposure, residential density, access to destinations) and walking is accumulating, few studies have investigated the relationship between the built environment and diabetes (self-reported or clinically-measured) [16]. These studies have reported that people living in more walkable neighbourhoods are less likely to report cardiometabolic risk factors that are closely associated with diabetes [17,18], whereas the presence of healthy food stores and restaurants [19], sport and recreation venues has been negatively associated with Type 2 diabetes [19]. However, to our knowledge, no studies have considered direct associations between other discrete environment features, such as the topography (hilliness), with Type 2 diabetes [16]. It is plausible that neighbourhood slope may be associated with Type 2 diabetes risk, with physical activity behaviours being the most likely pathway.

Variations in ‘slope’, otherwise known as incline (i.e., how steep or flat the ground is) is recognised as an important feature in urban design and landscape guidelines [20] because it can enhance landscape dynamics and pleasantness [21,22] but this is rarely adhered to in practice because it is easier to build housing on flat terrain. However, it appears that variations in slope may be good for health and policies to build on flat terrain may therefore have unintended consequences for good health. Notably, physical activity interventions in workplace environments have attempted to encourage the use of stairs instead of elevators for increasing physical activity levels [21,23,24]. Studies in controlled laboratory environments report that stair climbing requires 8.6 times *more* energy than the resting state, and even higher rates have been reported in field settings (9.6 METS; [25,26]). This energy expenditure is 7.6 times more than the energy expended while walking slowly on level ground (2.0 METS; calculations based on estimations outlined in the Compendium of Physical Activities in [27]). Similar to stair climbing, walking up hills is a physiologically vigorous physical activity [28] and can contribute to weight control [26]. For example, hilliness has been associated with higher exercise intensity, and more energy expenditure [26,29,30], and these in turn might reduce Type 2 diabetes risk [31]. Moreover, Eves and colleagues [26] suggest that because energy is expended by raising one’s weight against gravity, the speed at which one climbs is of minor importance and poor cardiovascular fitness need not impact the potential health benefits gained [26].

However, in free-living outdoor environments, studies suggest that for areas with greater slope, people are less likely to walk or cycle than those living in flatter neighbourhoods [32,33], indicating that steep hills (perceptions and objectively-measured) may be physical environmental barriers to walking [34]. Indeed, a Canadian study suggested that university staff and students reported the presence of sloping terrain was less attractive for walking and cycling [35]. This is likely due to the increased difficulty of - and thus greater energy expenditure required to – walk up or down steeper slopes. However, others have found the presence of hills to be positively associated with physical activity [36,37]. A US study for example, found that the presence of hills increased the odds of physical activity by 26% [37]. The investigators suggested hilly areas may be related with more scenic locales.

Nevertheless, given the additional benefits of walking up steeper slopes for physical activity levels (e.g., higher exercise intensity and more energy expenditure), greater neighbourhood slopes may also provide a protective barrier to weight gain, and therefore help prevent Type 2 diabetes onset in adulthood. Thus, this study explored whether living in ‘hilly’ neighbourhoods was associated with diabetes prevalence among the Australian adult population. As the pathway in which slope may influence diabetes is through physical activity behaviours, we hypothesised that given equal amounts of walking, and adjustment for other confounders, those who walk up hills rather than on flat surfaces are less likely to have diabetes.

## Methods

### Study participants and setting

This study forms part of the Life Course Built Environment and Health (LCBEH) project, a cross-sectional data linkage study exploring associations between built environment features and health across different life stages in Perth, Western Australia [38]. Perth is the state’s capital city with an urban population of approximately 1.7 million, which is 75% of the state [39]. Perth is isolated, sprawls some 170 km along the coast, has a relatively high standard of living with a Mediterranean climate, and is considered as having a ‘flat’ terrain relative to other Australian capital cities.

Participants were a stratified random sample drawn from the Perth metropolitan area who completed the Western Australian Health and Wellbeing Surveillance System (HWSS) survey from 2003–2009 (n = 21,347) administered by the Department of Health of Western Australia (DoHWA). For survey participants who gave permission for linkage to other datasets, built environment and (accessible) destinations data were calculated. Overall 74.7% consented to data linkage and had a geocoded home address (n = 15,954). Adults ≥ 25 years were included (n = 11,406). Ethics approval was obtained from DoHWA and The University of Western Australia (ref 2010/1).

### Self-reported diabetes (outcome variable)

Self-report of prior medically diagnosed diabetes (Has a doctor or nurse ever told you that you had diabetes?; no, yes) was obtained from the HWSS survey. The survey did not specify the type of diabetes (e.g., gestational, Type 1, Type 2), however, it is more likely that adults over 25 years of age will have Type 2 diabetes [2].

### Built environment features

It is necessary for participants’ ‘neighbourhood’ to be spatially defined, in order to measure the built environment within it [40]. Geographic Information Systems (GIS) software (ArcGIS v10) was used to operationalise the ‘neighbourhood’ using the road network service area at 1600 m around each participant’s home [41-43]. A 1600 m service area is typically used in studies with Australian adults [41-43], as this represents how far they could walk from home at moderate to vigorous intensity within 15 minutes, which is half of the recommended level of daily physical activity for adults [44]. Detailed information on the methods used to develop built environment variables is published elsewhere [38]. Briefly, a series of scripts were used to compute built environment measures using PYTHON v2.6 [45], a scripting software compatible with ArcGIS v10.

#### Slope (independent variable)

Slope measures the on-ground terrain or topography. Using the spatial analyst tool in GIS, Digital Elevation Model (DEM) data with a cell size of 90 m × 90 m were used to calculate slope values (percent slope where 0 represents flat and 100% represents 45 degrees) for the Perth metropolitan region. The mean of this slope measure was calculated for all cells that intersected the road network in each participant’s 1600 m service area using zonal statistics. The mean slope was used as a measure of hilliness or amount of terrain in the service area (i.e., overall slope of neighbourhood, which may not necessarily reflect the slope of potential or actual walking routes).

#### Other built environment variables (adjustment variables)

Distance to various destinations within 1600 m of each participant’s home was computed. Count of, and closest road network distance to, destinations within 1600 m of each participant’s home were computed using a script based on the Origin–destination (OD) Cost Matrix tool in ArcGIS v10. Destinations data were obtained from a variety of sources and re-classified into eight categories (i.e., parks. retail, health services, recreation, fast food/takeaway, larger food outlets, restaurants/cafes/coffee, other food destinations; see footnotes of Tables 1 and 2). These destinations were chosen as they have previously been shown to be associated with Type 2 diabetes [19,46].

**Table 1:** Characteristics of Life Course Built Environment and Health participants and their neighbourhoods, Perth, Western Australia (2003–2009)

	**Total sample (n = 11,406)**	**People with self-reported diabetes (n = 964)**	**People without self-reported diabetes (n = 10,442)**	***p*-value**
**Individual characteristics**				
Age in years (mean ± SD)	55.8 ± 15.8	64.3 ± 13.4	55.0 ± 15.7	<0.001
Sex - Male (%)	51.4	49.4	40.7	<0.001
- Female (%)	58.6	50.6	59.3	
Education				<0.001
**-** Mid-secondary (%)	8.8	16.3	8.1	
**-** Upper secondary (%)	20.3	22.0	20.2	
**-** Final year of secondary school (%)	11.1	8.1	11.3	
**-** Tafe/trade qualification (%)	36.3	38.7	36.1	
**-** University degree or equivalent (%)	23.5	14.9	24.3	
Income (AUD)				<0.001
**-** Under $20,000 (%)	19.6	35.2	18.2	
**-** $20,001-$40,000 (%)	23.3	29.2	22.8	
**-** $40,001-$60,000 (%)	16.0	12.7	16.3	
**-** $60,001-$80,000 (%)	14.3	7.7	14.9	
**-** Over $80,000 (%)	26.7	15.2	27.8	
Daily serves of vegetables usually consumed (mean ± SD)	3.0 ± 1.6	3.0 ± 1.6	3.0 ± 1.6	0.530
Daily services of fruit usually consumed (mean ± SD)	1.8 ± 1.2	1.9 ± 1.1	1.8 ± 1.2	0.163
Walking (mins in the last week) (mean ± SD)	130.4 ± 161.0	116.2 ± 166.3	131.7 ± 160.5	0.006
**Neighbourhood characteristics**				
Slope mean (mean ± SD)	3.3 ± 1.7	3.0 ± 1.5	3.3 ± 1.7	<0.001
Slope mean (tertiles) (%)				
**-** Tertile 1 (< 2.4)	33.3	41.1	32.6	<0.001
**-** Tertile 2 (2.4 – 3.7)	33.3	31.3	33.5	
**-** Tertile 3 (>3.7)	33.3	27.6	33.9	
Connectivity Z score (mean ± SD)	−0.02 ± 1.0	−0.04 ± 0.9	−0.02 ± 1.0	0.490
Residential density Z score (mean ± SD)	0.00 ± 0.9	−0.01 ± 0.9	0.00 ± 1.0	0.817
Land Use Mix Z score (Recreation) (mean ± SD)	0.01 ± 1.0	0.03 ± 1.0	0.01 ± 1.0	0.517
Parks (mean ± SD)	4.46 ± 6.4	4.1 ± 6.0	4.5 ± 6.4	0.040
Retail destinations (mean ± SD)	11.2 ± 14.6	10.9 ± 13.0	11.3 ± 14.7	0.489
Health service destinations (mean ± SD)	24.9 ± 42.2	21.8 ± 39.2	25.2 ± 42.4	0.019
Recreation destinations (mean ± SD)	1.7 ± 2.6	1.6 ± 2.4	1.7 ± 2.6	0.204
Fast food/Takeaway destinations (mean ± SD)	13.0 ± 17.0	12.6 ± 15.6	13.0 ± 17.1	0.450
Healthy food destinations (mean ± SD)	0.90 ± 1.3	0.8 ± 1.2	0.9 ± 0.3	0.075
Larger food outlet destinations (mean ± SD)	1.6 ± 1.8	1.5 ± 1.7	1.6 ± 1.8	0.140
Restaurant/Café/Coffee destinations (mean ± SD)	4.4 ± 9.5	3.9 ± 8.8	4.5 ± 9.5	0.081
Other food destinations (mean ± SD)	5.2 ± 5.8	5.3 ± 5.5	5.2 ± 5.8	0.916

Slope mean is percent slope.

Unless otherwise specified, all variables are continuous; SD = Standard Deviation.

Destinations: Count of destinations within 1600 m of participants’ homes (road service area):

**-Parks**: parks and green spaces.

**- Retail destinations:** Bike shop, Book shop, CD/DVD stores, Craft/Art supplies, Florist, General store, Gifts novelties souvenirs, Hardware store, News agent, Office stationery, Pharmacy, Photo shop, Post office, Sports store, Toys hobbies, Video store, Shopping centres, Pharmacy.

**- Health service destinations:** Doctor, Medical centre.

**- Recreation destinations:** Sports fields, Recreation centre, Swimming pool.

**- Fast food/Takeaway destinations:** Fast food, Take away food.

**- Healthy food destinations:** Health foods, Fruiterers and green grocers, Organic products, Vitamin products.

**- Larger food outlet destinations:** Shopping centres, supermarkets and groceries, markets**.**

**- Restaurant/Café/Coffee destinations:** Restaurants, cafes, coffee shops.

**- Other food destinations:** Bakers, Butchers, Cake/Pastry shops, Confectionery, Delicatessens, Seafood shops, Food delicacies, Hotels/Pubs, Halal products, Icecream, Petrol stations, Road houses, Food and general stores.

**Table 2:** Adjusted odds ratios for association of neighbourhood slope with self-reported diabetes: Life Course Built Environment and Health project, Perth, Western Australia (2003–2009)

		**Level of adjustment**				
**Neighbourhood network slope measure**	**Unadjusted**	**Model 1 (socio-demographic)**	**Model 2 (Model 1 + neighbourhood walkability)**	**Model 3 (Model 2 + destinations)**	**Model 4 (Model 3 + nutrition)**	**Model 5 (Model 4 + walking)**
		**OR (95% CI)**	**OR (95% CI)**	**OR (95% CI)**	**OR (95% CI)**	**OR (95% CI)**
Slope mean (continuous)	0.89 (0.85-0.93)	0.89 (0.85-0.94)	0.89 (0.84-0.93)	0.89 (0.83-0.94)	0.89 (0.83-0.94)	0.87 (0.80-0.94)
Slope mean (tertiles)	1.00	1.00	1.00	1.00	1.00	1.00
2 vs 1	0.74 (0.63-0.87)	0.75 (0.63-0.89)	0.75 (0.63-0.89)	0.78 (0.63-0.98)	0.78 (0.62-0.97)	0.72 (0.55-0.95)
3 vs 1	0.65 (0.55-0.76)	0.65 (0.54-0.78)	0.65 (0.54-0.78)	0.64 (0.51-0.80)	0.63 (0.51-0.80)	0.52 (0.39-0.69)

OR: Odds ratio; CI: Confidence Interval.

Model 1: Socio-demographic variables = age, gender, education, income.

Model 2: Model 1 + Neighbourhood walkability variables = Connectivity Z score, Residential density Z score, Land Use Mix Z score (Recreation).

Model 3: Model 2 + destinations.

Model 4: Model 3 + nutrition (daily number of vegetables and fruits consumed).

Model 5: Model 4 + walking (minutes of walking in the past week).

Destinations: Count of destinations within 1600 m of participants’ homes (road service area):

**-Parks**: parks and green spaces.

**- Retail destinations:** Bike shop, Book shop, CD/DVD stores, Craft/Art supplies, Florist, General store, Gifts novelties souvenirs, Hardware store, News agent, Office stationery, Pharmacy, Photo shop, Post office, Sports store, Toys hobbies, Video store, Shopping centres, Pharmacy.

**- Health service destinations:** Doctor, Medical centre.

**- Recreation destinations:** Sports fields, Recreation centre, Swimming pool.

**- Fast food/Takeaway destinations:** Fast food, Take away food.

**- Healthy food destinations:** Health foods, Fruiterers and green grocers, Organic products, Vitamin products.

**- Larger food outlet destinations:** Shopping centres, supermarkets and groceries, markets.

**- Restaurant/Café/Coffee destinations:** Restaurants, cafes, coffee shops.

**- Other food destinations:** Bakers, Butchers, Cake/Pastry shops, Confectionery, Delicatessens, Seafood shops, Food delicacies, Hotels/Pubs, Halal products, Icecream, Petrol stations, Road houses, Food and general stores.

The standardised z-scores for three other built environment variables typically known to be associated with health [14] were adjusted for in analyses: 1) land-use mix (area in km^2^ of land use types calculated according to an entropy formula adapted from that originally used by Frank et al. [42,47]); 2) street connectivity (ratio of number of three-way or more intersections to area in km^2^); and 3) residential density (ratio of number of dwellings to residential area in hectares).

### Socio-demographic variables

A core set of variables adjusted for in analyses included: sex (male; female), age in years (continuous), and indicators of socio-economic status (SES) such as education attainment (≤mid-secondary; upper secondary; final year of secondary school; TAFE/trade qualification; university degree or equivalent) and annual household income (≤AUD$20,000; AUD$20,001-$40,000; AUD$40,001-$60,000; $AUD60,001-$80,000; >AUD$80,000).

### Self-reported behaviour variables

#### Self-reported nutrition

Daily serves of fruit usually consumed and daily serves of vegetables usually consumed were the nutrition variables asked in the HWSS survey and adjusted for in previous LCBEH studies [18,48]. A ‘serve of fruit’ was defined as equal to one medium piece of fruit, two small pieces of fruit, or one cup of diced fruit. A ‘serve of vegetables’ was described as equal to half a cup of cooked vegetables or one cup of salad.

#### Self-reported amount of walking

Self-reported total minutes of walking continuously for at least 10 minutes, for recreation, exercise or to get to or from places in the last week was obtained from the HWSS survey, and truncated at 840 minutes per week based on previous Australian studies [49,50].

### Statistical analyses

SPSS v19 for Windows was used for analyses. Pearson’s chi-square tests and Independent t-tests were used to examine the association between diabetes and socio-demographic characteristics (age, sex, education, income), self-reported nutrition and walking behaviour, neighbourhood walkability variables (residential density, connectivity, land-use mix), destinations, and neighbourhood terrain slope (Table 1). The mean slope was analysed both as a continuous and a categorical variable (tertiles). Logistic regression models were used to estimate the association between neighbourhood slope mean (continuous and tertiles: reference category = lowest) and self-reported medical diagnosis of diabetes after successive adjustment for several classes of potential confounding variables: socio-demographic variables (model 1), neighbourhood walkability (model 2), destinations (model 3), nutrition (model 4), and amount of walking (model 5, final model). Interactions between walkability variables and slope were explored by including the interaction in the models. Analysis of variance (ANOVA) was used to explore whether participants living in neighbourhoods with greater slopes walked more or less than those living in flatter neighbourhoods. Values of *p <* 0.05 were considered statistically significant.

## Results

### Slope

**Figure 1:**
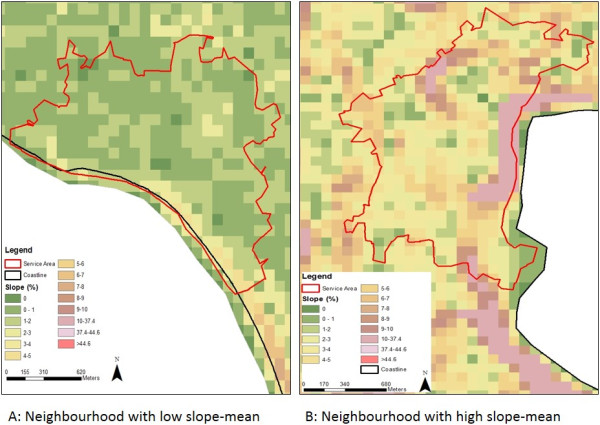
**Two examples of neighbourhood slope cell values for Western Australian adults in the Life Course Built Environment and Health Project (2003-2009). (a)** Neighbourhood with low slope-mean (e.g., 0.3). **(b)** Neighbourhood with high slope-mean (e.g., approximately 11).

**Figure 2:**
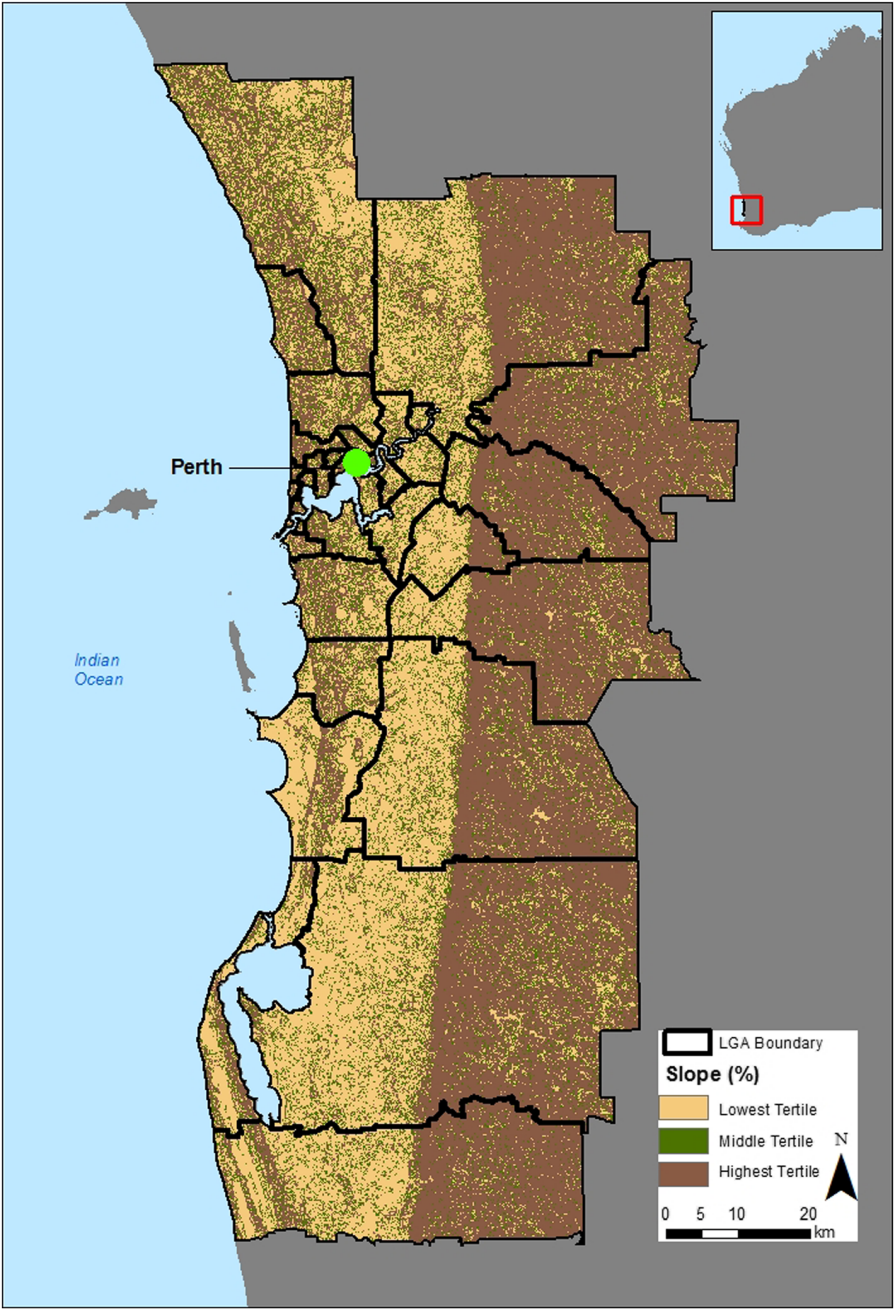
**Location of low, medium, and high slope-mean areas for adults in the Life Course Built Environment and Health project (2003–2009), Perth, Western Australia.** LGA Boundary = Local Government Association Boundary.

The mean slope ranged from 1 to about 10 for most services areas (not reported here) and Figure 1 illustrates the slope measure for two service areas, one with a low slope mean (1a) and one with a high slope mean (1b). Figure 2 shows the spatial variation in slope mean values across the participant sample (low, medium, high slope). There appears to be clustering in some areas, however, the highest tertile slopes (dark brown areas) are typically located in the Darling ranges (Perth’s hills to the east), the western suburbs, and neighbourhoods along the coast, north of the City.

### Participant characteristics

A total of 964 adults (8.5%) reported a prior medical diagnosis of diabetes. Table 1 represents the characteristics of participants and their neighbourhoods for the total sample and by diabetes status. There were significant demographic differences (age, sex, education, income) between adults with and without diabetes (*p* < 0.001). There were no significant associations between having diabetes and fruit consumption (*p =* 0.163) or vegetable consumption (*p =* 0.530). Adults without diabetes reported 15.5 more minutes of walking in the last week than those with diabetes (*p =* 0.006). There were few significant differences in neighbourhood characteristics, except for parks (*p =* 0.040) and health service destinations (*p =* 0.019) for those with and without diabetes; however, there was a highly significant difference in both slope mean (*p <* 0.001) and slope tertile distribution (*p <* 0.001) between adults with and without diabetes. The neighbourhoods of those with diabetes had less sloping terrain than the neighbourhoods of those without diabetes (*p <* 0.001). We found that those living in neighbourhoods with slope in the highest tertile slightly walked more (188 mins) than those in the middle (179 mins) and lowest (173 mins) quartiles (overall ANOVA *p =* 0.003).

### Adjusted associations between slope and self-reported diabetes

The statistically significant negative association between slope mean/slope tertile and self-reported diabetes persisted after progressive adjustment for socio-demographic variables, neighbourhood walkability measures, destinations, nutrition variables and walking behaviour (Table 2). Indeed, there was little variation or attenuation in the estimated odds ratios through the progressive adjustment modelling process. In the fully adjusted model (Model 5), the odds ratio for self-reported diabetes was 0.72 (95% CI 0.55-0.95) and 0.52 (95% CI 0.39-0.69) for adults living in neighbourhoods with moderate (tertile 2) and higher levels (tertile 3) of slope, respectively, compared with adults living in neighbourhoods with the lowest levels of slope (tertile 1). Similarly, in the continuous slope model 5, the odds of having diabetes was 13% lower (odds ratio 0.87; 95% CI 0.80-0.94) for each increase of one percent in mean slope. This association remained constant despite adjustment for sociodemographics, neighbourhood walkability variables, nutrition measures, and minutes of walking. We also tested for interactions (results not reported here) between walkability variables and slope to explore whether the association with slope was modified by walkability variables, and none were significant.

## Discussion

The results suggest a strong relationship between self-reported diabetes and absolute levels of neighbourhood slope. Greater neighbourhood slope was associated with lower odds of self-reported diabetes. The odds of self-reported diabetes for adults living in neighbourhoods with slope in the highest tertile were about 50% lower than the odds of adults living in neighbourhoods with slope in lowest tertile. This raises the question as to whether the slope of a neighbourhood is a key risk factor for diabetes or whether this is a spurious association that can be explained through confounding by other factors.

As physical inactivity is a risk factor for Type 2 diabetes, it is therefore likely that our observed associations are due, at least in part, through the effect of the neighbourhood environment factors on physical activity in general, and amount of walking in particular. Our observed association between slope and diabetes remained after adjustment for neighbourhood walkability variables (street connectivity, residential density and mix of land uses), access to destinations and self-reported minutes of walking. Our walking variable was the total self-reported minutes of walking in the last week and was not specific to the neighbourhood; therefore it is possible that the association between slope and diabetes may have attenuated if we had adjusted for a measure of walking in the neighbourhood. However, as can be seen in Table 2, there was little attenuation from Model 4 to Model 5 when we adjusted for minutes of walking. As such, either our control for walking was inadequate, or it would therefore appear that neighbourhood slope is an independent protective factor for diabetes. It could be, for example, that all walking is not equal i.e., that walking in hilly neighbourhoods is more beneficial because it is more intense than walking in flat neighbourhoods [26].

Walking in neighbourhoods with steeper slopes may offer additional opportunities for expending energy, even if the walking speed is not particularly fast [26]. Thus, greater neighbourhood slopes may provide a protective barrier to weight gain, and in doing so, protect against Type 2 diabetes onset. This may be particularly important for those who are overweight or obese, those who have no other choice but to walk and cycle, or those who do not engage in moderate-vigorous forms of physical activity for recreation purposes. In fact, overweight individuals’ walking in environments with greater slopes immediately benefit, because they do more work against gravity than those of normal weight [26]. Moreover, walking to and from destinations (i.e., walking for transport to get to places), particularly in a hilly neighbourhood may help some individuals achieve sufficient levels of physical activity, particularly those who cannot or do not participate in other types of physical activity [51]. Over time, these small incidental increases could have a significant benefit. For example, a previous study speculated that the energy expenditure of walking up and down two flights of stairs every day for 1 year amounts to 2.7 kg for an 80 kg man [52]. Moreover, stair climbing or walking in hilly areas provides a form of weight-bearing exercise, which can strengthen the musculoskeletal system, thus helping to further decrease the likelihood of developing chronic health conditions [53]. However, neighbourhoods with steeper slopes may also discourage people from walking [32,33], particularly those who experience weight problems, are physically unfit, or physically impaired (e.g., a physical disability, those with conditions that limit walking such as arthritis and osteoporosis) [34]. Thus a possible explanation for our observed cross-sectional association is that people with conditions that put them at higher risk of diabetes have chosen to live in non-hilly areas.

On the other hand, adults who choose to engage in more moderate-vigorous physical activity may incur additional benefit. Other studies have suggested that the presence of hills is associated with recreational physical activity [36,37]. It may be that people that walk, cycle, jog, or run up slopes for *recreation* purposes have the opportunity to create additional challenges for themselves, and to experience the added benefit of more intense exercise.

These results have important implications for policy and practice. When building new neighbourhoods, developers tend to flatten the terrain because it is easier and more economical to build housing on flat terrain [54] compared with hilly terrain. Flattening neighbourhoods may have unintended consequences by potentially reducing benefits of walking in two ways: 1) it may make the neighbourhood less interesting and reduce the amount of recreational walking; and 2) the walking that is undertaken in flatter neighbourhoods may be less intense than walking undertaken in hilly neighbourhoods. As we have demonstrated, this may have negative health consequences. However, as previous findings on slope and physical activity behaviours are mixed, caution must be exercised as it may also be that people are more likely to walk in flat areas, particularly older adults [32], and those with other physical conditions likely to limit walking behaviour such as physical disability. Nevertheless, further studies are required to confirm our findings.

Our study has several limitations. As adults of low SES are more at risk of being overweight or obese [55-58], and developing Type 2 diabetes [55,59], other possible explanations for our observed association include inadequate adjustment for diet-related factors and/or for socioeconomic factors. For example, a study by Sayeed et al. (2004) found that compared with non-tribal populations in Bangladesh, a tribal population had a higher prevalence of diabetes despite living in Khagrachari, a primarily *hilly* area northeast of Bangladesh [60]. Thus, living in a hilly location did not protect people from diabetes onset. Other factors more predictive of diabetes incidence may come into play (e.g., genetic predisposition, lower access to health care, lower education). Indeed, genetic predisposition, diet-related factors, and socioeconomic factors are widely researched and established risk factors of Type 2 diabetes [1]. Nevertheless, we have accounted for potential confounders available to us. In our study, high neighbourhood slope was spatially clustered along the northern coast and in the eastern hills which typically include more affluent suburbs. However, we have adjusted for individual education and household income and so we expect residual confounding by SES to be small. As we hypothesized that the most likely pathway in which slope influences diabetes is through physical activity (e.g., amount of walking), other potential confounders include other forms of exercise (e.g., leisure-time physical activity). Future research may also look to exploring *intensity* of walking in hillier vs. flatter neighbourhoods by using accelerometer-measured physical activity, or weighting minutes of walking in the neighbourhood according to the amount of increase in neighbourhood slope. As our walking measure was not neighbourhood-specific, it is unknown how much of their total walking was done in their neighbourhood. To this end, a 1600 m ‘neighbourhood’ buffer does not represent participants’ complete activity spaces as they may travel, and subsequently be exposed to, built features beyond their ‘neighbourhood’ to undertake their daily activities (e.g., work, shopping, entertainment) [40,61]. We could not adjust for the time spent living in the neighbourhood; this may be an important adjustment variable for health and place-based studies because residents who have lived in a hilly neighbourhood for longer may be more exposed to greater slopes and vice versa. Our diabetes outcome variable was not specific to Type 2 diabetes as the survey did not specify this; however, given that the average age of adults with diabetes was almost 65 years, it is likely that most will have Type 2 diabetes [3]. Finally, there is the possibility of selection bias in our sample as built environment variables were not available for one-quarter of the survey participants (those who did not consent to linking their survey data to other databases). The non-linked sample was of similar age and sex distribution to those included in the study except that they were slightly younger and more likely to be female. They were more likely to have completed the highest level of education. There were no differences in household income (see [48]).

Both the mean and standard deviation of slope were initially explored. The mean slope describes the absolute level of slope of the neighbourhood, while the standard deviation of slope captures the heterogeneity in the distribution of slope. However, given that Perth generally has a ‘flat’ terrain, there was a high correlation between slope-mean and slope-standard deviation (r = 0.93; not reported here). Moreover, the crudeness of the slope calculation (i.e., 90 m × 90 m cells) suggests that it is more a measure of hilliness of the general service area (i.e., neighbourhood) rather than the road network *per se* because the slope measure of a road network cell is determined by the change of elevation with all surrounding cells not just those in the same direction as the road. Therefore, we have interpreted slope-mean and slope-standard deviation to essentially measure the same construct and have used slope-mean as a measure of ‘hilliness’ or amount of sloping terrain. As the crudeness of the slope measure (i.e., 90 m × 90 m of all cells intersecting the road network) may not provide an accurate measure of slope along the road network, finer-grained DEM data cells are required for future studies or slope measured along GPS-measured (Geographic Positioning System) actual routes are required. To this end, a slight difference in slope may be small for a single road segment, however, our slope measure represents the average slope over the neighbourhood, and as such this change in average slope represents a more substantive, but still modest, change in hilliness. Future work in this field should be tested in cities with greater variation in hilliness across areas. Neighbourhood slope may also be correlated with *other* environmental features such as neighbourhood greenness. For example, hilly areas may be greener, and the presence of greenery may influence factors along the causal pathway to diabetes (e.g., physical activity and obesity) [16]. It is also possible that the strength of the relationship between neighbourhood slope and diabetes might vary across space, and datasets with geocoded addresses for participants might use geographically weighted logistic regression to explore this further. Moreover, our cross-sectional study design means that it still remains to be proven that neighbourhood slope is a key independent risk factor for the development of diabetes.

## Conclusions

This study is unique and suggests an association between the hilliness of a neighbourhood and the level of diabetes. It could be that living in a hilly neighbourhood is protective or that this finding is spurious. We attempted to control for confounding with all available data, and as the relationship remained, a plausible mechanism may be the effect of hilliness on exercise intensity. This question needs to be explored in large-scale community studies, especially cohort/longitudinal studies, before neighbourhood slope could be confidently regarded as having a protective effect on the development of Type 2 diabetes in adults. Nevertheless, the results are promising and raise questions about the practice of flattening land in new housing developments.

## Abbreviations

CI: Confidence Interval; DEM: Digital Elevation Model; DoHWA: Department of Health of Western Australia; GIS: Geographic Information System; HWSS: Health and Wellbeing Surveillance System; LCBEH: Life Course Built Environment and Health; LGA: Local Government Association; MET: Metabolic Equivalent of Task; SA: Service area; WA: Western Australia.

## Competing interests

The authors declare that they have no competing interests.

## Authors’ contributions

KV developed the first draft of the manuscript. MK, FB, and BGC contributed to the conception and design of the study. MK provided statistical advice. SH computed the GIS measures and developed the figures. MK, JK, SH, SF, HB, AN, FB and BGC provided feedback on the manuscript’s contents and approved the final submission. All authors read and approved the final manuscript.

## Authors’ information

KV is a Research Fellow at the McCaughey VicHealth Centre for Community Wellbeing at The University of Melbourne, but conducted the research at the Centre for the Built Environment and Health, The University of Western Australia. MK is a Professor of Biostatistics at the School of Population Health, The University of Western Australia. JK is a Research Fellow at Baker IDI Heart and Diabetes Institute and the McCaughey VicHealth Centre for Community Wellbeing, The University of Melbourne. SH is a GIS Research Assistant at the Centre for the Built Environment and Health, The University of Western Australia. HB is a Senior Research Fellow at the McCaughey VicHealth Centre for Community Wellbeing, The University of Melbourne. AN is a Research Fellow at The Queensland University of Technology. SF is a Research Assistant Professor at the Centre for the Built Environment and Health, The University of Western Australia. FB is a Professor of Public Health and the Director of the Centre for the Built Environment and Health, The University of Western Australia. BGC is a Professor of Public Health and the Director of the McCaughey VicHealth Centre for Community Wellbeing, The University of Melbourne.
